# Family composition and age at menarche: Findings from the international Health Behaviour in School-aged Children study

**DOI:** 10.1186/s12978-019-0822-6

**Published:** 2019-12-05

**Authors:** Martin Steppan, Ross Whitehead, Juliet McEachran, Candace Currie

**Affiliations:** 1 School of Medicine, University of St Andrews, St Andrews, UK; 2 Glasgow Caledonian University (GCU), London, UK; 3 NHS Health Scotland, Edinburgh, UK; 4 National Youth Mental Health Foundation, Melbourne, Australia; 5 Psychiatric University Hospital Basel, Basel, Switzerland

**Keywords:** Age at menarche, Psychological and psychosomatic problems, Family structure, Body mass index, Life history theory, Pubertal timing

## Abstract

**Background:**

Early menarche has been associated with father absence, stepfather presence and adverse health consequences in later life. This article assesses the association of different family compositions with the age at menarche. Pathways are explored which may explain any association between family characteristics and pubertal timing.

**Methods:**

Cross-sectional, international data on the age at menarche, family structure and covariates (age, psychosomatic complaints, media consumption, physical activity) were collected from the 2009–2010 Health Behaviour in School-aged Children (HBSC) survey. The sample focuses on 15-year old girls comprising 36,175 individuals across 40 countries in Europe and North America (*N* = 21,075 for age at menarche). The study examined the association of different family characteristics with age at menarche. Regression and path analyses were applied incorporating multilevel techniques to adjust for the nested nature of data within countries.

**Results:**

Living with mother (Cohen’s d = .12), father (d = .08), brothers (d = .04) and sisters (d = .06) are independently associated with later age at menarche. Living in a foster home (d = −.16), with ‘someone else’ (d = −.11), stepmother (d = −.10) or stepfather (d = −.06) was associated with earlier menarche. Path models show that up to 89% of these effects can be explained through lifestyle and psychological variables.

**Conclusions:**

Earlier menarche is reported amongst those with living conditions other than a family consisting of two biological parents. This can partly be explained by girls’ higher Body Mass Index in these families which is a biological determinant of early menarche. Lower physical activity and elevated psychosomatic complaints were also more often found in girls in these family environments.

## Plain English summary

The age of menarche is a reliable marker of pubertal timing in girls. Early age of menarche has been found to be associated with several negative psychological and physical health outcomes in later life, e.g. cardiovascular disease, all-cause mortality, breast cancer, type 2 diabetes, obesity, gynaecological, obstetric, gastrointestinal, musculoskeletal, and neuro-cognitive disorders, early initiation of risk behaviours and teenage pregnancies. In the literature, a so-called “stepfather-effect” has been described suggesting that girls who live with a stepfather hit puberty significantly earlier than girls who do not. The causes of this effect are still unclear.

In this study, we used cross-sectional self-report data from the 2009–2010 Health Behaviour in School-Aged Children (HBSC) study to test the presence of such an effect in a large international dataset. Including data of more than 36,000 15-year old girls, the presence of a “stepfather-effect” has clearly been corroborated, although a “stepmother-effect” was even more pronounced. The strongest puberty-accelerating effect was found for girls who live in a foster home or with someone else. Vice versa, the presence of biological mother and father and siblings was related to later menarche. No link was found between the onset of menarche and whether a girl lives with her grandparents or not.

The study also investigated, if these effects can be explained by some simple lifestyle differences between traditional families and other family compositions (media consumption, physical activity, Body Mass Index): For example, it is well established, according to Mendelian Randomization studies, that higher Body Mass Index (BMI) is related to earlier puberty in girls. One explanation could be that the families with step-parents simply have different eating behaviors, thereby causing the difference. We could indeed find that girls who live with step-parents, in a foster home or with someone else have higher BMI, lower physical activity, more media consumption, and higher psychosomatic complaints, indicating more sedentary behavior. Up to 89% of the effect of different family compositions can be explained when controlling for these lifestyle differences.

In conclusion, family composition matters when it comes to pubertal timing. However, it needs to be said that although statistically significant, the effects are small. For example, Body Mass Index has a ten-fold higher impact on the age of menarche. Also the country in which the girls live plays a five times more important role than family composition. Nonetheless, even when controlling for these lifestyle variables, the “stepfather-effect” could not be fully explained, leaving room for future research.

## Background

Age at menarche is considered to be reliable indicator of pubertal timing [1], especially when reported soon after its occurrence [2]. It is a critical event in girls’ pubertal development representing a biological, psychological and social transition within their developmental trajectory [3]. Its timing has implications for many aspects of health and well-being both during adolescence and later in life. In adolescence, early menarche in girls has been associated with mental health problems, e.g. depression, eating disorders and body dissatisfaction [4]. Menarcheal timing has been associated with behavioural problems, e.g. substance use, early initiation of risk behaviours, including sexual behaviour or teenage pregnancy [5]. In later life, early menarche has been associated with a wide range of somatic risks and conditions, e.g. cardiovascular disease, all-cause mortality especially among smokers, breast cancer, type 2 diabetes, obesity, gynaecological, obstetric, gastrointestinal, musculoskeletal, and neuro-cognitive disorders [6]. The secular decline in age at menarche charted by various studies in recent years [7] could therefore be understood as a cause for public health concern.

Several factors have been identified as determinants of early menarche including biological, psychological and genetic factors: high body mass index [8], high caloric diet, psychological and social distress, exposure to environmental estrogens have been associated with earlier pubertal timing [9]. One area which has received much attention is the family context in which girls are growing up. Consistent evidence has been found showing that the absence of a biological father accelerates pubertal timing in girls [10]. There is, however, inconsistent evidence on whether the presence of a step-father has an independent effect [11]. In general, there is evidence that broader family disintegration can accelerate pubertal timing in girls [12]. In addition to family structure other characteristics of the immediate social environment have been found to advance pubertal timing, e.g. low socioeconomic status [13], exposure to violence [14] and physical or sexual abuse [15]. Considering these findings, early pubertal timing appears to be a phenomenon related to detrimental social circumstances, and might therefore have wider implications for public health.

Various mechanisms have been proposed to explain how the social environment can influence pubertal timing, of which many are based on evolutionary theory. “Life History Theory” is an evolutionary framework which predicts that harsh life circumstances will lead to a faster reproductive strategy, i.e. earlier childbearing and a greater quantity of offspring and lower parental investment, which is a trade-off against fewer children and higher investment per child [16]. This framework has also been used to understand the occurrence of teenage pregnancy, high number of offspring and lower life expectancy in areas of socioeconomic deprivation [17]. Another evolutionary argument hypothesizes that father presence leads to late puberty as a phylogenetic mechanism to avoid inbreeding [18]. A further complex pathway has been proposed which involves the transmission of an androgen receptor gene between father and daughter. This gene putatively leads both to early maturation in the daughter and promiscuous behaviour in the father, which subsequently produces a father absence effect without a direct causation [19].

Within the range of approaches in this research field, several limitations must be considered. Firstly, many studies into family characteristics and their potential impact on age at menarche have been conducted with US samples, and a smaller number with Canadian, European or Australian national samples [10]. Considering the US, that has one of the highest incidences of single parent or stepparent families, analyses covering a larger cultural variation in family types would be informative. Secondly, the importance of specific family members, and in particular of father absence versus stepfather presence, is not entirely clear within the existing literature. In most circumstances where a stepfather is living in the household the biological father is absent. Research is needed in order to disentangle these effects and to control for statistical complications. Thirdly, despite meta-analytic and genetic evidence, exact pathways remain unknown with respect to how family composition may affect pubertal timing in real life [10] [20] [21]. Regardless of evolutionary mechanisms, a physiological mechanism must give rise to the observed effects between family structure and pubertal timing.

This study seeks to address some of the limitations outlined above. It uses a data set from multiple countries across Europe and includes the US and Canada stemming from the Health Behaviour in School-Aged Children Study (HBSC) which includes great diversity in family types from 40 large representative national samples [22]. As well as data on the paternal context – father absence and stepfather presence, it provides rich data on other aspects of the family structure – presence or absence of mother, presence of stepmother, presence of siblings and grandparents. It also records information on girls growing up with no family members – living in foster homes and children’s homes. Additionally, data are available on other domains that pertain to the theoretic framework. These include self-reported age at menarche, psychological measures (psychosomatic complaints), physical measures (Body Mass Index), and related behavioural measures (physical activity, sedentary behaviour) [23]. The analysis presented uses this unique data set to examine the association between different family structures and age at menarche.

## Methods

### Research design

This study is a cross-sectional observational study based on the 2009–2010 Health Behaviour in School-Aged Children (HBSC) survey [23]. Nationally representative random samples of 11-year-old, 13-year-old and 15-year-old children were available including a total sample size of *N* = 213,595 (including boys) in 41 countries and regions in Europe, the Middle East and North America. A detailed description of the International Health Behaviour in School-Aged Children Study (HBSC) can be found at the survey’s website www.hbsc.org.

### Sample

Data on the age at menarche, family structure and covariates were collected from the 2009–2010 Health Behaviour in School-Aged Children (HBSC) survey were clustered into samples of 11-year-old, 13-year-old and 15-year-old children. Considering previously reported overall age at menarche between 12 and 13 years in western countries [7], a large proportion of girls in the age groups of 11- and 13-year-olds have not had their first period yet. This would involve an increasing negative skew of post-menarcheal girls in these age groups and a significant deviation from normality. Hence, only girls from the 15-year-old age group were selected in order to obtain a more comparable sample, and to eliminate cohort effects, leading to a sample of *N* = 21,094 girls in 36 countries that provided data on the age of menarche. The sample also included 4% 14-year-old and 6% 16-year-old girls. This is due to the sampling procedure in the HBSC survey, where classes are sampled and not specific individuals to achieve representativity, where some individuals can be slightly deviating from the target age because of earlier or later schooling, pupils who have to repeat a year or other circumstances.

### Measures

#### Age at menarche, missing data and coverage

Within the 41 countries and regions that participated in the 2009–2010 Health Behaviour in School-Aged Children (HBSC) survey, data from 36,175 15-year old girls were available. Response levels varied by different variables and countries. Data on age at menarche, were not available for Armenia, Lithuania, Russia and Turkey. The response levels for this variable ranged from 25.2% for girls in Israel to 92.1% in Ukraine. The overall rate of valid data on the age at menarche was 58.3% representing a sample of *N* = 21,094. Data for Spain had to be excluded due to non-normality of the distribution indicating a data coding problem. For all other variables non-response rates varied between 1.5% for psychosomatic complaints and 13.4% for Body Mass Index.

#### Family measures and covariates

Family characteristics were assessed through binary variables (0 = No, 1 = Yes) whether a certain family member lives in the “main home” with the respondent. This format was used in the HBSC questionnaire for mother, father, grandmother, grandfather, stepmother and stepfather. Two more binary variables assessed whether the respondent lives in a “foster / child home”, and whether they lived with “someone or somewhere else”. The number of brothers and sisters living in the main home with the respondent was assessed as two separate integers and recoded into binary variables (no brother / sisters versus one or more). As covariates / mediators, age, BMI, media consumption, physical activity, family affluence and psychosomatic complaints were used. For physical activity, media consumption, family affluence and psychosomatic complaints composite scores were used based on principal component analyses (PCA). The first principal component of these measures was used instead of the average score due to better psychometric properties. Family affluence is a composite measure which represents a well-established indirect assessment of family material wealth and socioeconomic background- the HBSC Family Affluence Scale (FAS) [24, 25].

### Statistical analysis

#### Cross-country differences and clustering within countries

To show which countries stood out on which variables, cross-country differences on all variables in the study were calculated using the standardised deviation of a country’s mean compared to the mean of all other countries (Table 1). For each variable also an Interclass-Correlation Coefficient (ICC) was calculated in order to quantify the clustering of the variable within countries (Table 1). High ICC’s indicate that individuals in a country are similar to each other, indicating the necessity of multilevel techniques. ICC was calculated with R using the package “multilevel” and the function “ICC1” using “country” as the cluster variable [26].

**Table 1:** Sample description and variables included

	Sample Size	Age	Age at menarche	Father at home	Mother at home	Step-Father at home	Step-Mother at home	Grand-Father at home	Grand-mother at home	Brothers at home	Sisters at home	Some-one else at home	Foster home	Psychosomatic complaints	Media consumption	Phsical Activity	Body Mass Index	Family Affluence
Unit	n	years	years	**%**	%	%	%	**%**	**%**	Mean	Mean	%	%	Perc.R.	Perc.R.	Perc.R.	BMI	Perc.R.
Armenia	517	15.49		**92.1+**	98.0	0.8	0.6	**32.9+++**	**50.0+++**	0.85	0.89	0.3	0.5	45.6	59.6	44.8	19.9	18.9
Austria	935	15.35	12.94	75.1	93.7	6.4	0.8	11.3	15.8	0.79	0.77	2.1	0.7	36.7	55.4	46.2	20.8	56.5
Belgium flemish)	546	15.45	12.85	70.4	88.3	13.1	5.4	5.4	7.3	0.87	0.80	1.4	0.6	40.9	53	48.1	20.4	54.5
Belgium (french)	672	15.46	12.84	68.5	92.3	13.2	3.4	2.7	3.2	1	0.99	1.4	0.9	52.8	54.4	44.9	20.3	56.4
Canada	2756	15.45	12.72	69.9	88.4	10.1	3.4	3.6	5.6	0.78	0.85	3.2	1.4	52.8	53.3	58	21.7	59.2
Switzerland	1108	15.35	12.95	77.9	95.5	7.6	2.6	4.8	7.0	0.87	0.87	1.2	0.8	48.4	40.6	47.3	20.4	55.6
Czech	775	15.45	12.95	68.7	95.1	14.7	1.4	12	17.3	0.77	0.35-	0.5	0.6	58.3	55.4	46.2	20.8	38.0
Germany	904	15.39	12.97	78.9	94.8	9.5	1.9	8.6	12.6	0.82	0.75	1.9	0.6	42.1	56.5	52.4	20.7	61.6
Denmark	649	15.65	12.93	68.5	87.7	9.9	3.4	0.7	1.1	0.91	0.88	1.5	0.8	38.6	60.8	49.2	20.4	61.5
Estonia	737	**15.78+**	13.14	64.0	93.8	14.6	1.6	7.6	14.9	0.7	0.71	4.3	0.4	48.8	60	41.4	20.8	48.0
England	624	15.59	12.81	64.8	89.5	9.8	1.9	3.0	5.7	**1.65++**	**1.71+++**	2.1	0.4	51.2	57	45.6	20.4	55.6
Estonia	1041	15.46	13.14	83.6	93.8	3.9	0.7	4.8	9.3	0.64	0.60	2.0	0.8	42.9	49.7	40.7	21.0	50.1
Finland	1102	15.68	12.81	75.5	92.8	10	3.4	1.3	2.2	0.96	0.92	1.8	1.1	52.0	45.3	51.3	21.0	61.2
France	1002	15.46	12.82	73.7	93.3	10.7	3.0	2.6	3.9	0.88	0.88	1.5	1.1	54.8	50.7	37	20.2	61.0
Greenland	208	15.46	12.71	**57.5-**	**78.6---**	8.6	2.3	4.7	5.8	1.38	1.27	0.0	**9.5+++**	39.4	**38.5-**	40.7	21.6	18.6
Greece	806	15.66	12.74	85.5	95.9	2.2	1.1	6.1	13.2	0.81	0.72	6.7	0.4	54.8	57.8	39.4	21.6	45.1
Croatia	1227	15.49	12.94	88.1	96.5	3.1	0.6	15.6	27.7	0.77	0.73	1.9	0.4	46.0	58.1	37.1	21.2	38.5
Hungary	934	15.48	12.91	73.1	93.2	8.8	1.4	6.1	12.9	0.79	0.78	2.3	0.8	56.4	58.8	42.8	20.8	34.9
Ireland	733	15.45	12.97	75.8	89.3	6.3	1.5	2.8	5.7	1.3	1.21	1.7	1.2	48.8	**37.6-**	48.7	20.7	51.9
Israel	671	15.68	13.01	87.7	95.9	2.8	1.3	2.7	5.2	**1.56+**	**1.42+**	1.0	1.9	**69.5+**	68.5	30.4	21.0	53.1
Iceland	1807	15.47	12.98	73.4	94.5	12.6	2.3	1.5	2.1	1.02	0.93	5.3	0.6	55.6	49.1	55	21.3	73.7
Italy	782	15.45	**12.31---**	82.5	93.7	2.5	0.4	7.6	13.3	0.75	0.69	1.2	0.9	64.1	50.1	33.3	21.0	49.0
Lithuania	847	15.67		69.6	94.9	9.2	0.6	10.2	22.5	0.68	0.65	0.0	1.8	50.4			20.5	30.8
Luxembourg	680	15.46	12.89	76.2	94.6	9.4	2.4	3.5	6.7	0.94	0.89	2.4	1.1	54.8	53.6	54.2	21.0	63.1
Latvia	709	15.58	13.19	61.1	93.3	14.4	1.9	7.8	21.2	0.75	0.63	2.3	0.5	48.4	55.5	47.2	20.3	35.8
Macedonia	722	15.46	13.06	88.5	92.2	0.8	0.4	**26.7++**	**34.8+**	1.2	1.24	0.7	1.7	39.7	69.3	41.2	20.7	27.3
Netherlands	727	15.45	13.00	83.2	96.5	5.3	1.9	0.6	1.2	1.43	1.37	0.7	0.1	**34.8-**	61.2	**61.6+**	20.1	62.0
Norway	628	15.51	12.94	73.2	91.0	11.7	4.1	3.2	4.2	0.99	0.97	5.4	1.3	46.8	58.9	53.8	21.0	77.8
Poland	725	15.68	13.01	83.3	96.1	4.2	0.9	11.4	22.1	0.91	0.83	3.8	0.6	54.0	64.1	40.7	20.8	34.6
Portugal	873	15.46	**12.52-**	79.5	93.7	6.4	1.9	6.1	10.4	0.71	0.72	**8.6+**	0.3	36.3	59.7	32.8	21.2	54.7
Romania	956	**15.10---**	13.00	79.7	95.3	4.5	0.5	12.7	20.6	0.63	0.59	2.8	1.9	57.1	69.7	34.3	20.7	24.0
Russia	928	15.42								0.79	0.77			44.4	62.1	39.2	20.0	28.7
Scotland	1335	15.54	12.72	70.0	92.2	9.4	1.6	3.0	4.1	0.88	0.86	2.2	0.4	50.0	60.5	50.3	20.7	57.3
Sweden	1031	15.49	12.79	78.0	92.0	5.6	**11.1+++**	1.0	1.4	1.07	1.08	0.7	1.1	56.0	58.9	50.1	20.6	67.8
Slovenia	901	15.6	12.87	83.6	95.7	5.4	1.3	16.9	25.5	0.75	0.73	4.8	0.4	**33.0-**	47.4	40.6	21.4	60.1
Slovakia	953	15.35	12.84	77.2	92.5	6.0	1.2	12.1	21.3	0.75	0.76	4.2	0.8	49.6	60.3	51.4	20.5	35.9
Turkey	902	**15.79+**		87.0	94.8	0.6	0.9	5.5	11.4	1.34	1.27	2.4	0.5	**67.7+**	53.4	**27.2-**	20.5	8.80-
Ukraine	1016	15.71	13.10	71.9	92.8	8.3	0.7	12.6	28.1	0.66	0.61	3.8	0.3	54.4	45.2	42.7	20.4	17.6
USA	924	15.46	12.63	61.4	87.6	13.6	4.2	4.7	8.3	1.08	1.03	**8.6+**	0.8	51.6	40.3	49.2	**22.7+++**	54.4
Wales	782	15.7	12.70	65.9	90.0	12.3	2.1	2.6	4.0	0.88	0.84	**9.5++**	1.0	46.0	58.3	46.6	21.4	54.3
Valid N	36,175	36.175	21.094	35.021	35.070	34.937	34.926	34.812	34.847	32.293	31.966	33.907	34.943	35.635	32.719	32.719	31.329	34.343
TOTAL^a^	23,949	15.51	12.87	75.5	92.81	7.91	2.05	7.23	12.24	0.93	0.89	2.71	1.03	49.39	55.09	44.71	20.81	47.45
ICC		0.144	0.02	0.031	0.018	0.021	0.018	0.055	0.09	0.047	0.05	0.017	0.007	0.009	0.038	0.039	0.029	0.196

*Note.* Cross-tabulation by region, Intra-class correlation (ICC), Significant differences (bold) +/− (*p*<,05) ++/−− (*p*<,01) +++/−−− (*p*<,001) (+ indicate above average, − below cross-regional average)

^a^For sample size the total is the sum of all cases, For all other variables total represents the cross-regional average (can deviate slightly from the individual average), Perc,R, represents percentiles calculated from the average standardized factor score

#### Pairwise comparisons (univariate analyses)

**Fig. 1:**
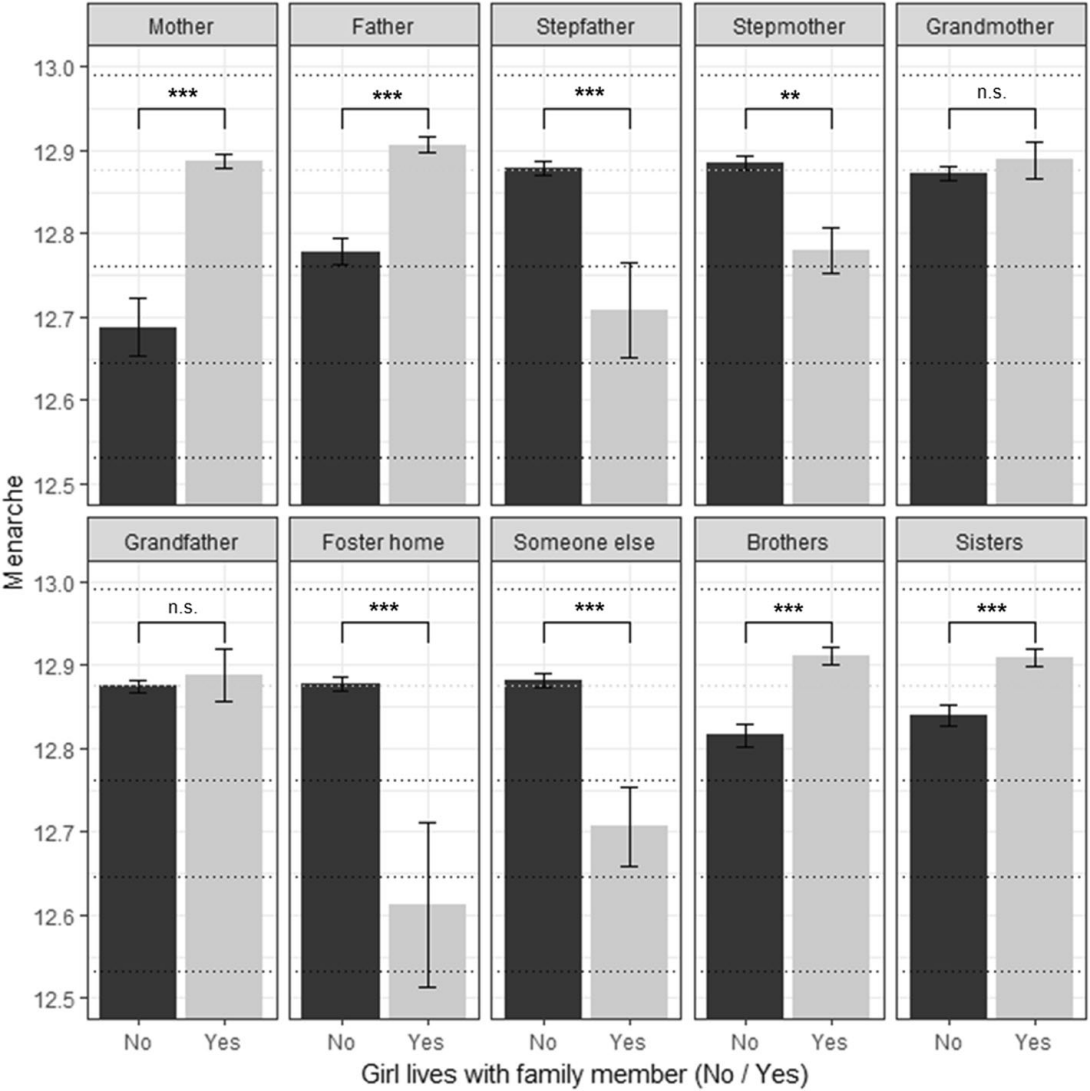
Univariate Differences in Pubertal Timing by Family Member. Age at menarche for different family members living in the main home with the child (yes / no). Total sample grand mean M = 12.81, SD = 1.14 [years]); asterisks represent significance levels of t-tests (*** *p* < .001; ** *p* < .01; n.s. = not significant); whiskers represent standard errors of the mean. Distance between horizontal dotted lines represents a tenth of a standard deviation (0.114 years)

Pairwise comparisons (Fig. 1) were applied to test differences between different family members living with the girl and age of menarche, regardless of potential confounding variables (e.g. BMI). The effect of each family member living with the respondent was tested using independent sample t-tests. Significance was adjusted for multiple testing using Bonferroni-correction. Due to different sample sizes for these comparisons the power of these t-tests were calculated using the R package “pwr” [27]. Effect sizes were calculated using the formula for Cohen’s d.

#### Regression analyses (multivariate analyses)

**Fig. 2:**
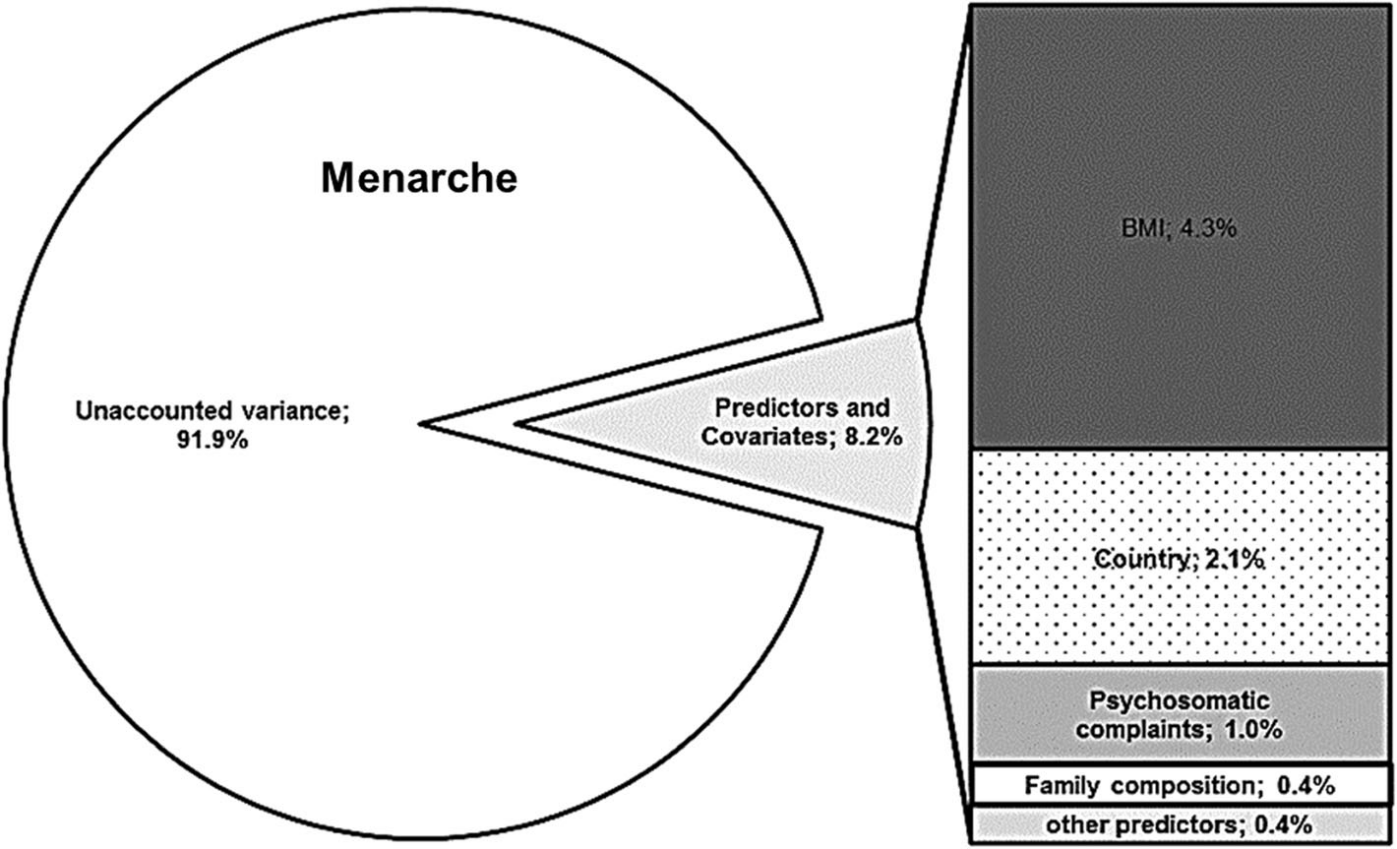
Variance accounted for by different predictors. Variance components of the age at menarche attributable to different predictors and unexplained variance (white). Results are based on simultaneous multiple linear regression

**Fig. 3:**
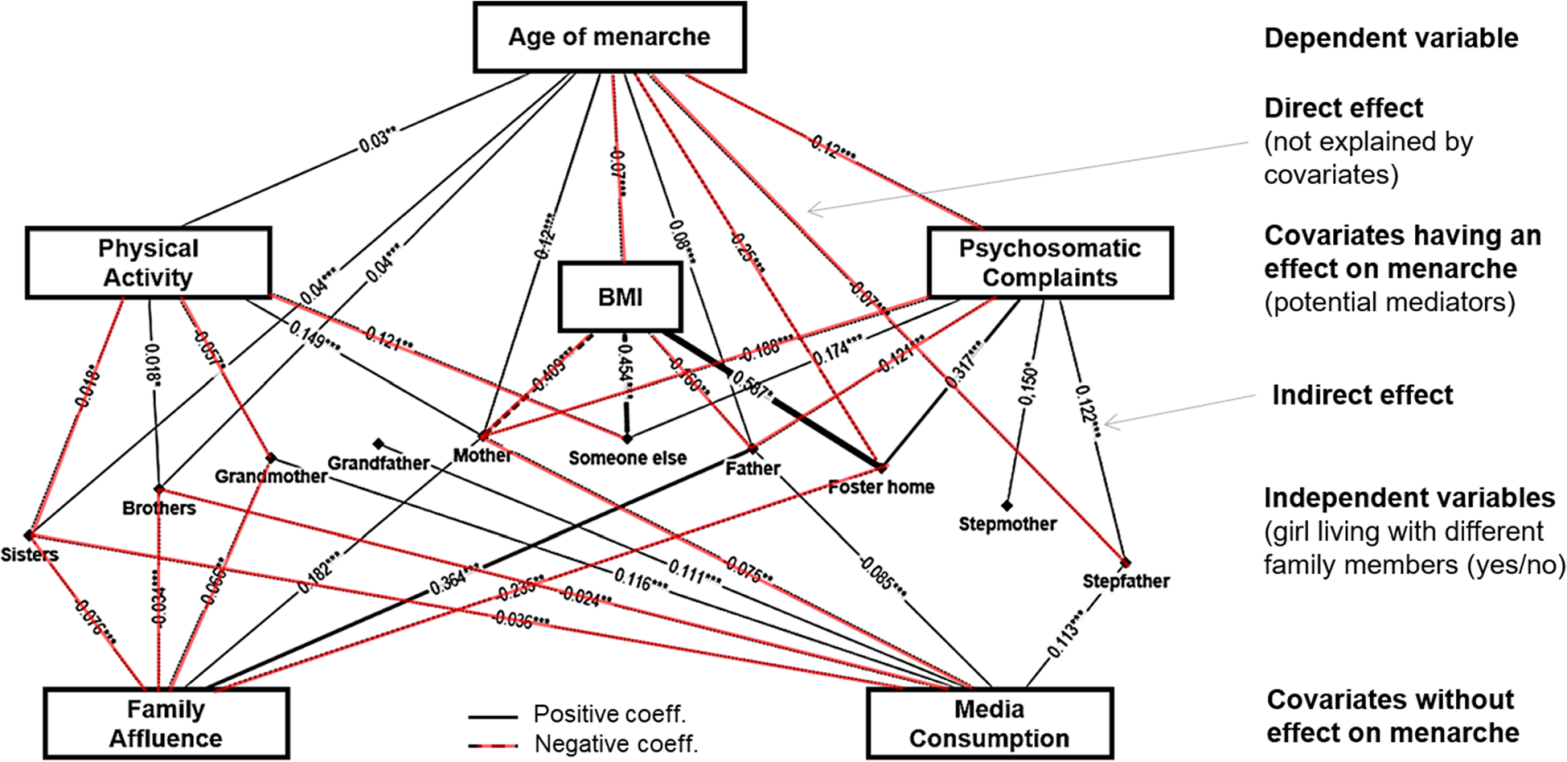
Composite Path Model. Comprehensive visualization of multiple path models associating family members (first layer) with covariates (second layer) and age of menarche (third layer). Visualization according to a network-analytical approach (Sugyama, Software: NodeXL). Solid lines represent positive associations, dotted lines negative assocations

Variance components (Fig. 2) were calculated to show how much of the percentage of variance of the age at menarche is accounted for by different variables when using standard multiple linear (simultaneous) regression using SPSS. Path models (Fig. 3, Table 2) represent a more complex type of regression analysis, where a whole network of associations and indirect effects can be tested. Path models were performed with R using the package “lavaan” [28]. Within these models the age at menarche and all covariates (except for age) were controlled for the country mean (“random intercept models”). In a second step, significant paths between the age at menarche and covariates (and among covariates) were modelled. In a third step, different family variables were modelled with respect to their direct association with age at menarche, and their indirect effects via covariates. This step was performed for each family variable separately due to high complementarity of family variables and the incurred risk of multicollinearity (i.e. if the respondent lives with her father, it is not very likely that there is also a stepfather present in the main home). Therefore, ten separate path models were calculated, one for each family member that the respondents could potentially live with. As a last step, all models were merged into one figural depiction using a network analytical approach using the Software NodeXL and a hierarchical Sugiyama layout which is well-suited for visualizing path and mediation models [29]. A sensitivity analysis for path models was carried out using a more homogeneous sample of girls who had their first period between the age of 12 and 13, and mother and father living in the main home of the respondent.

**Table 2:** Summary statistics for multilevel mediation models corresponding to Fig. 3

	DIRECT AND INDIRECT EFFECTS				ASSOCIATION WITH COVARIATES (INDIRECT EFFECTS)						GOODNESS OF FIT		
Family Member	Indirect	Direct	ProportionIndirect / Direct		Age (p1)	Media (p2)	Psych.(p3)	BMI (p4)	FAS (p5)	Physical (p6)	CFI	TLI	SRMR
Mother	0.055***	0.122***	45%		−0.012*	−0.075**	− 0.188***	− 0.409***	0.182***	0.149***	1	1.009	0.0006
Father	0.024***	0.075***	32%		−0.024**	− 0.085***	− 0.121***	− 0.160**	0.364***	0.029	1	1.004	0.0012
Stepmother	−0.030*	−0.063	47%		0.002	0.039	0,150*	0.143	0.106	−0.064	1	1.011	0.0003
Stepfather	−0.016*	−0.066*	25%		−0.001	0.113***	0.122***	−0.014	−0.013	− 0.039	1	1.011	0.0005
Grandmother	−0.008	−0.033	24%		−0.003	0.116***	0.009	0.048	−0.066**	−0.057*	1	1.011	0.0005
Grandfather	−0.006	−0.017	39%		−0.010	0.111***	−0.011	0.062	−0.037	−0.03	1	1.012	0.0005
Foster home	−0.083***	−0.251*	33%		0.001	−0.058	0.317***	0.587*	−0.235**	−0.158	1	1.011	0.0005
Someone else	−0.055***	−0.062	89%		0.008*	0.009	0.174***	0.454***	−0.055	−0.121**	1	1.011	0.0005
Brothers	0.001	0.041***	2%		0.026	−0.024**	0.015	0.021	−0.034***	0.018*	1	1.011	0.0005
Sisters	0.002	0.044***	4%		0.038	−0.036***	0.012	0.019	−0.076***	−0.018*	1	1.009	0.0009

*Note.* Beta weights for columns 1,2,4–9. *CFI* Comparative Fit Index, *TLI* Tucker Lewis Index, *SRMR* Standardized Root Mean Residual. * *p* < .05; ** *p* < .01; *** *p* < .001 (bold). *BMI* Body Mass Index, *Media* media consumption, *FAS* Family Affluence Scale, *Physical* Physical Activity, *Psych*. psychosomatic complaints

## Results

### Sample description & cross-national differences

Table 1 illustrates the included variables in the study, and the cross-country distribution of these variables. Significant variations from the cross-country average were observed for several variables and countries. The age at menarche was significantly lower in Italy and Portugal. The age of the sample, although highly homogeneous, was significantly higher in Estonia, and significantly lower in Romania. In Greenland, the percentage of both mothers and fathers in the main home of respondents was significantly lower than in other countries. Stepmothers in the main home were significantly more frequently observed in Sweden. Grandmothers and grandfathers were more frequently reported to live in the main home of girls in Macedonia. The number of siblings living in the main home of the respondent was significantly higher in England and Israel. ‘Living with someone else or somewhere else’ was most frequently reported in Wales and Portugal. Significantly more respondents in Greenland lived in a household without their biological parents. Psychosomatic complaints were significantly higher in Israel and Turkey, whereas lower in the Netherlands. Media consumption was significantly lower in Greenland and Ireland. Physical activity was highest in the Netherlands and lowest in Turkey. A significantly higher BMI was observed in the United States. Family affluence was lowest in Turkey. Table 1 illustrates samples sizes and the age at menarche for different family variables in this study. Intraclass correlations (ICC) showed that only family affluence was clustered within countries, whereas for the other variables the within-country variation was higher than the between-country variation.

### Univariate associations between family members and age of menarche

Figure 1 shows the average age at menarche by different family members living with the respondent (yes = black or no = gray). The lowest age at menarche was observed for girls who live in foster homes (M = 12.61 years; SD = 1.2), followed by girls who live with someone else in their main home (who is not a close family member; M = 12.71; SD = 1.21). Girls who lived with a stepfather or stepmother also exhibited a lower average age at menarche (M = 12.81; SD = 1.18, M = 12.71; SD = 1.20, respectively). Girls who live with their mother (M = 12.89; SD = 1.14) or father (M = 12.91; SD = 1.14) had a later age at menarche. The presence of brothers and sisters (both M = 12.91, SD_sisters_ = 1.14; SD_brothers_ = 1.15) was also significantly associated with higher age at menarche compared to girls who do not live with siblings in their main home. The presence of grandmothers or grandfathers (both M = 12.89) was not significantly associated with the age at menarche. Considering the small standard errors of the means (vertical lines in Fig. 1), significant effects can be recognized by non-overlapping lines. Significant differences remain after controlling for multiple testing (Bonferroni). The statistical power and effect sizes in terms of Cohen’s d were: Mother (d = .12; Power: 78%), Father presence (d = 0.07; Power: 95%), sister presence (d = 0.04; Power: 37%), brother presence (d = 0.06; Power: 74%), living in a foster home (d = −.16; Power: 9%), living with someone else (d = − 0.11; Power: 26%), stepfather presence (d = − 0.06, Power: 13%), stepmother presence (d = − 0.10; Power:26%). Positive d values indicate later age at menarche and negative earlier age at menarche.

### Multivariate analyses of age of menarche, family members and covariates

Figure 2 shows variance components of the age at menarche explained by different variables using multiple linear regression. The biggest part of the variance of age at menarche remains unexplained (91.85%). Only 8.15% of the age at menarche can be explained by predictors examined in this study. The most important of these predictors is BMI explaining 4.32% of the variance. Country and region explain 2.1% of the variance. Psychosomatic complaints account for 0.96% of the variance in the age at menarche. Family composition, age and other predictors together account for less than 1% of the variance. In the following, we are showing how this 0.4% of variance related to family composition affect age at menarche in more detail, and which explanatory pathways indicate why different family compositions are associated with different pubertal pace in girls.

Table 2 shows direct and indirect effects for each family variable. The strongest indirect (beta = −.083, *p* < .001) and direct effect (beta = −.251,*p* < .05) was calculated for living in a foster / child home which were strongly associated with earlier age at menarche. A considerable percentage (33%) of this direct effect can be explained by the association with increased BMI (beta = .587, p < .05) and increased psychosomatic complaints (beta = .317; p < .001) for girls living in foster homes. All of these paths indirectly explain the lower age at menarche for these girls, because these variables show corresponding associations with the age at menarche (higher BMI and higher rates of psychosomatic complaints are associated with lower age at menarche). Causality as a predictor had been proven at least for BMI in previous studies [30]. Similar paths were observed for the presence of mother and father: both are associated with lower BMI and lower psychosomatic complaints, which are both associated with later age at menarche (through direct or indirect pathways). The effects of a stepfather/ stepmother can be explained in part by increased psychosomatic complaints which are associated with earlier menarche. The highest proportion of an indirect effect was observed for “living with someone else” (89%). This means that nearly all of the full direct effect can be explained by the higher BMI, higher psychosomatic complaints and lower physical activity in these respondents. For the number of brothers and sisters, almost no indirect effect was found, although direct effects were highly significant for both. This means that 98% of the effect for brothers, and 96% of the effect for sisters likely have reasons beyond those covariates used in this model. All models showed very good model fit (CFI = 1.000; TLI > 1.000; SRMR<.001).

Figure 3 combines all path models in Table 2 into one composite visualisation using a network-graph based approach. Paths between covariates and the age at menarche remain constant for all models irrespective of which family variable was included in the model. The strongest predictor of the age at menarche were psychosomatic complaints, which were associated with a lower age at menarche (beta = −.12, *p* < .001). Further significant associations were observed for the following: BMI, associated with a lower age at menarche (beta = −.07, p < .001); physical activity, associated with later age at menarche (beta = .03, p < .001).

## Discussion

This study showed significant associations between family composition and age at menarche of 15-year old girls. If considered in terms of process then it appears that there is a menarche-delaying effect of families consisting of two biological parents and siblings and accelerating effects of family compositions which differ from this pattern. Although family characteristics account only for a small part of the variance directly (< 1%), a variety of highly significant indirect paths could be identified, which were stable at a cross-country level, based on large sample sizes indicating robust effects of family composition on pubertal timing.

Our findings are in line with a previous study corroborating the menarche-accelerating effect of an absent biological father [31]. Findings relating to a stepfather-effect [32] which were found in another earlier study were less pronounced in this study. Several reasons may play a role, for example, one reason may be that fluctuations in the family structure cannot be adequately depicted through a point assessment of family structure at age 15. The presence of a stepfather and the absence of a father are by nature negatively correlated, which can cause collinearity problems in regression analyses if considered simultaneously [33]. The findings in this study also corroborate an earlier report on menarche-delaying effects of siblings [18]. Some authors have argued that this might be due to reduced nutrition in families with many children [34] – a mediation path which could not be identified via BMI in our study (we found an effect of BMI itself, but not from siblings to BMI). The most pronounced effect in this study was a menarche-accelerating effect of living in a foster home. This effect was more than twice as high as the menarche-delaying effect of living with the biological mother and three times as high as living with the biological father. Considering that children in foster homes are not living with their biological parents, and other adults look after them, the total effect may resemble a combination of a “presence of step-parents-“ and “absence of biological parents“-effect. The results emphasize the previously reported influence of environmental risk factors and sexual maturation in this group [35].

Path analyses showed that considerable parts of these effects can be attributed to intervening covariates. The key role of BMI as a tertiary variable corresponds with earlier findings and the menarche-accelerating effect of higher BMI [36]. Using Mendelian Randomization, BMI had been verified as a likely causal agent of early menarche [30]. Since family composition is also not affected by back-causation (i.e. family composition is usually not a consequence of the child’s behavior), significant paths we identified via BMI, are potentially causal mediation paths. Paths related to BMI are in logical correspondence with the observed family effects. Girls who live with their biological parents have significantly lower BMI, whereas girls in foster homes, and those who live with someone else have significantly higher BMI. This protective effect of living with two biological parents against obesity is in line with previous research and corroborates a mediation path of lower media consumption in these families which may be related to less sedentary behaviour [37]. The mediating effect of BMI for girls who live with ‘someone else’ is so pronounced that together with increased psychosomatic complaints and lower physical activity in these girls it accounts for 89% of the variance and completely outperforms a direct effect. This finding suggests that the effect of living with ‘someone else’ is almost completely explained by the covariates used in this study. Another previously unestablished confounder in this study was psychosomatic complaints. Similar to BMI, psychosomatic complaints are lower in families with a biological mother and father, whereas significantly higher in families with step-parents and in foster homes and for girls who live with someone else. Based on the evident association of psychosomatic complaints with age of menarche, it may resemble a confounding variable. The relationship between early puberty and mental health problems had been known from earlier literature [4], but had been seen by some authors as a transitory problem during puberty [38]. A key question of these path analyses is whether psychosomatic complaints or increased BMI are a cause or a consequence of puberty [8]. Earlier research and evidence on puberty indicate that changes in metabolism occur with puberty [39] and there is energy conservation in females’ bodies potentially leading to higher BMI if physical activity does not increase. Similarly, psychological wellbeing has often been linked to sex hormones [40] which may suggest that psychosomatic complaints are a consequence of early puberty rather than vice versa. HBSC data are not able to fully elucidate this question, since longitudinal data could give more accurate answers to cause-consequence mechanisms. However, BMI had been verified as a likely causal agent of early menarche [30]. Hence, paths from family composition via BMI to age at menarche, can be considered very likely causal mechanisms. Performed sensitivity analyses (using a very homogeneous sample of girls living with biological mother and father who had their period between the age of 12 and 13) showing a same size effect of psychosomatic complaints and BMI on the age at menarche, also provide support for the hypothesis that BMI and psychosomatic complaints may be significant predictors, definitely significant correlates of pubertal timing.

These findings on psychosomatic complaints corroborate the hypothesis that psychological factors, and stress in particular, play a role in the understanding of environmental effects on pubertal timing and are also partly in line with conceptions based on a life-history-approach [41]. These findings contradict earlier research which found that stress in childhood was not related to the age at menarche [42]. Regardless of evolutionary theories, there needs to be a biological mechanism to understand how such an effect happens in vivo. Several biological explanations for how stress and psychological impairment could affect pubertal timing may be considered: (i) stress may be accompanied by higher secretion of stress hormones and higher activity of organs related to stress, e.g. adrenal glands and the HPA-axis (hypothalamic-pituitary-adrenal-axis) [43]. One predecessor of puberty is related to higher activity of these glands, a stage also referred to as adrenarche [44]. A higher activation of the HPA prior to puberty may trigger adrenarche by the system regulating pubertal timing. This link may also explain the association of early puberty and further behavioral problems which have been linked to HPA [45]. (ii) stress can accelerate biological ageing [46] and therefore lead to early puberty based on yet unknown “receptors” in the body which detect biological age and start the cascade of sexual maturation, e.g. through telomeres [47].

Our findings pertaining to family structure are partly consistent with a life history theory approach, which proposes that accelerated pubertal timing under harsh environmental conditions constitutes an adaptive trait [48]. On the other hand, no explanation via family affluence was seen, which would be predicted by this framework. Our findings, however, suggest that not living with both biological parents has a greater impact on pubertal timing than does material wealth. One conclusion we might draw, given the effect of psychosomatic complaints, is that social aspects of the home interact with biological development more than material aspects. The findings give rise for follow-up research to identify further mechanisms by which the effects of family composition can be explained.

### Limitations

Besides those already mentioned, general limitations of this study need to be considered: (i) results are based on self-reports of participants which may be subject to intentional and unintentional response biases [49]; (ii) several potential mediators and predictive variables were not included which may further elucidate the interplay of family characteristics and pubertal timing, e.g. quality of relationship with biological and step parents, timing of dissolution of biological family, sexual experience, alcohol and drug consumption etc. [50]; (iii) several non-linear effects may affect the data which was not controlled for in this study; (iv) girls who had not reached menarche at the age of 15 were excluded from the analysis. Due to data collection procedures, these cases could not be distinguished from girls who had experienced menarche but did not complete the question on timing of menarche, hence their percentage cannot be calculated. However, based on the average age at menarche for the total sample (M = 12.81) and the standard deviation (SD = 1.15) the age of 15 represents a z-score 1.90 indicating that - assuming a normal distribution of the age at menarche – about 2.8% of the sample (i.e. 590 girls) can be estimated being premenarcheal and therefore missing in the data. This theoretic right truncation (censoring) of the data represents a statistical problem that could not have been circumvented in this study, since no older age group than 15-year olds is included in the study.

## Conclusions and recommendations

The present study has shown that family composition plays a significant role in pubertal timing, and that the absence of biological parents and siblings is related to earlier age at menarche. These effects can in part be explained by lifestyle variables (BMI, physical activity, media consumption). The findings of this study support the need to acknowledge that family decomposition can have a measurable impact on reproductive ageing in girls. We found the same pattern of decomposed families also associated with increased levels of psychosomatic complaints. This highlights the importance to support affected adolescents in order to reduce the impact on their physical and mental health.

## Data Availability

According to the guidelines of *Springer Nature* the data used in this article can be downloaded at http://www.uib.no/en/hbscdata.

## Abbreviations

BMI: Body Mass Index
CFI: Comparative Fit Index
FAS: Family Affluence Scale
HBSC: Health Behaviour in School-aged Children Study
HPA: Hypothalamic-pituitary-adrenal-axis
ICC: Intra-class Correlation Coefficient
M: Mean
p: Significance level
R: Open Source statistical software R
SD: Standard deviation
SPSS: Statistical Package for the Social Sciences
SRMR: Standardized Root Mean Square Residual
TLI: Tucker-Lewis Index
